# Telomeres and longevity

**DOI:** 10.18632/aging.100437

**Published:** 2012-02-23

**Authors:** Pat Monaghan

**Affiliations:** Institute of Biodiversity, Animal Health & Comparative Medicine Graham Kerr Building, University of Glasgow Glasgow G12 8QQ

While the basic elements of telomere biology are very similar across many taxa [1], there is diversity among species, and among tissues, in telomere dynamics. Not surprisingly, given that species differ in many other relevant aspects of their biology, including the pattern of activity of the telomere-restoring enzyme telomerase simple comparisons of average telomere length across species do not map directly onto interspecific variation in maximum lifespan [2-4]. In relatively long-lived species, telomerase is downregulated in most somatic cells, thought to have evolved as a mechanism to counteract an increased risk of tumour formation, particularly in endotherms [1]. One potential cost of this is that tissue renewal capacity is limited, resulting in somatic deterioration with age. However, within a given long-lived species, there is good reason to predict that variation in average telomere length in somatic cells will be related to potential lifespan. Examining this link is fraught with difficulties, not least of which is that studies covering the entire lifespan of a cohort of long lived animals take a very long time. Circumventing this by looking at telomere length and subsequent survival in individuals that are already old omits all the individuals who died early in life (and who may have had the shortest telomeres). The alternative approach of comparing average telomere length in a cross sectional sample of individuals of different ages suffers from a related bias; mean telomere length could actually appear to increase with age if individuals with short telomeres die and so drop out of the sample. Furthermore, since telomere length is a dynamic character, if it is predictive of lifespan, we also need to know at what life history stage the relationship is strongest. Longitudinal studies are therefore essential.

We recently examined the relationship between telomere length and longevity in a longitudinal study of a small songbird, the zebra finch *Taeniopygia guttata* [5]. Longevity is a feature of avian life histories [6]. Like long lived mammals, zebra finches have low telomerase activity in somatic tissues [7]. We measured telomere length from 25 days of age onwards in a cohort of birds and recorded their natural lifespan, which varied from less than one to almost nine years. We found that there was indeed a highly significant relationship between lifespan and telomere length.

Importantly, it was telomere length in early life that was most predictive of lifespan. Because the longer lived individuals had relatively long telomeres even at 25 days, our data also clearly illustrate how, as the cohort got older, the surviving sample was increasingly biased towards those that started life with long telomeres.

This study raises a number of important questions. Telomere length measured towards the end of the growth period (i.e. at 25 days) was the strongest predictor of lifespan. This was true even if we excluded the third of birds with the shortest lifespans; if we included in the analysis measurements of telomere length taken both in early life and at adulthood, the latter was not significant. What processes are responsible for the differences among individuals in their telomere length at 25 days? Is it the ‘starting’ telomere length that really matters, to which variation in telomere loss later in life simply adds noise? Is this therefore an inherited characteristic? Or is it variation in nutritional conditions or stress exposure during growth, known to affect telomere loss [8], that is responsible for generating most of the variation in telomere length at 25 days?

The causal mechanisms that might link telomere length to life expectancy are generally thought to affect old individuals. Some of the birds in our study died before we might have expected them to be suffering from diseases generally associated with old age. But should we stop thinking that telomere length only matters when individuals are old? Our data suggest that short telomeres at any age are associated with an increased risk of death. We do not know the detailed causes of death of our birds, other than that they died of intrinsic causes (i.e. not infection, accidents, poor nutrition etc). But might the pathways linking telomere length and lifespan differ at different life stages? Might the relationship be driven by different factors early in life but causal later in life?

And how relevant are studies of birds to studies of humans? Are they more relevant than the typical comparisons with species that have evolved traits associated with short lifespans, like *C. elegans*, yeast or mice?
